# Wurster Fluidised Bed Coating of Microparticles: Towards Scalable Production of Oral Sustained-Release Liquid Medicines for Patients with Swallowing Difficulties

**DOI:** 10.1208/s12249-019-1534-5

**Published:** 2019-11-11

**Authors:** Valentyn Mohylyuk, Kavil Patel, Nathan Scott, Craig Richardson, Darragh Murnane, Fang Liu

**Affiliations:** 1 Department of Clinical and Pharmaceutical Sciences, University of Hertfordshire, Hatfield, AL10 9AB UK; 2 Fluid Pharma Ltd., Nexus, Discovery Way, Leeds, LS2 3AA UK

**Keywords:** Multiparticulates, Controlled release, Paediatric, Geriatric, Dysphagia

## Abstract

Suspension of microparticles in an easy-to-swallow liquid is one approach to develop sustained-release formulations for children and patients with swallowing difficulties. However, to date production of sustained-release microparticles at the industrial scale has proven to be challenging. The aim of this investigation was to develop an innovative concept in coating sustained-release microparticles using industrial scalable Wurster fluidised bed to produce oral liquid suspensions. Microcrystalline cellulose cores (particle size <150 μm) were coated with Eudragit® NM 30 D and Eudragit® RS/RL 30 D aqueous dispersions using a fluidised bed coater. A novel approach of periodic addition of a small quantity (0.1% *w*/w) of dry powder glidant, magnesium stearate, to the coating chamber *via* an external port was applied throughout the coating process. This method significantly increased coating production yield from less than 50% to up to 99% compared to conventional coating process without the dry powder glidant. Powder rheology tests showed that dry powder glidants increased the tapped density and decreased the cohesive index of coated microparticles. Reproducible microencapsulation of a highly water-soluble drug, metoprolol succinate, was achieved, yielding coated microparticles less than 200 μm in size with 20-h sustained drug release, suitable for use in liquid suspensions. The robust, scalable technology presented in this study offers an important solution to the long-standing challenges of formulating sustained-release dosage forms suitable for children and older people with swallowing difficulties.

## INTRODUCTION

Oral sustained-release dosage forms have valuable benefits in comparison with immediate release dosage forms: they allow the optimisation of pharmacokinetics, improve pharmacodynamics, and decrease dosing frequency, improving compliance and the general effectiveness of the treatment (1). Most sustained-release dosage forms are tablets, including matrix and coated tablets, or osmotic-pump systems; thus, they are not suitable for older people who have swallowing difficulties (2), including adults with dysphagia (3). A high proportion of older adults experience difficulties in swallowing solid dosage forms (4). Crushing tablets, which is commonly used to overcome swallowing problems (5), is not applicable for sustained-release tablets because it compromises their functionality, leading to dose dumping with undesirable side effects and even toxicity (6,7).

Liquid dosage forms, such as drops, solutions, syrups and suspensions, are suitable for patients with swallowing difficulties (8) but these cannot be easily formulated with a sustained-release profile. Several approaches have been attempted to produce sustained-release liquid medicines including suspensions of microparticles (MPs) *e.g.* drug-loaded ion-exchange resins (9), *in situ* gelling of liquids (10,11) and multiple-layer emulsions (12). Drug-containing MPs may have more reproducible gastric emptying profiles and smaller risks of dose dumping compared to non-disintegrating sustained-release tablets (13) but only a few marketed sustained-release liquid products are available. These utilize reconstitution of MPs based on ion-exchange resin complexation, for example amphetamine (Dyanavel™ XR), clonidine (Clonidine™ ER), methylphenidate (Quillivant™ XR), and hydrocodone/chlorpheniramine (Tussionex™ ER) (14–16). However, ionic-resin complexations are only applicable to ionisable (acidic or basic) drugs and require a complicated multi-step production process including additional polymer coatings to control drug diffusion rate (17).

Other methods of producing sustained-release MPs include alginate MPs prepared by calcium cross-linkage (18,19), spray-dried (20,21) and spray-congealed MPs (22,23) and MPs prepared using emulsion solvent evaporation (24). Wurster fluidised bed coating is routinely used in pharmaceutical processes. It offers an industrial-scalable method for producing drug-loaded discrete MPs surrounded by polymer film-coatings providing sustained drug release (25). The size of the MPs is crucial for the creation of effective and stable liquid dosage forms, influencing dosage uniformity (26), sedimentation rate (27), as well as oral sensations such as grittiness (28) and patients’ adherence to the medication (29). Recent investigations suggest that particles with a size of 250 μm or less are preferable in order to achieve patient compliance (28,30). However, coating particles of this size range using fluidised bed is a challenge because of the high tendency for particle agglomeration and aggregation (31,32). The limited ability of the widely used fluidised bed coaters to produce MPs smaller than 250 μm, especially using aqueous polymer dispersions, prevents their use to produce liquid sustained-release medicines.

The aim of this investigation was to develop an innovative concept in coating MPs using a Wurster fluidised bed to achieve robust manufacturing of sustained-release MPs that are suitable for use in oral liquid medicines.

## MATERIALS AND METHODS

### Materials

Metoprolol succinate was purchased from Sinobio Chemistry Co. Ltd. (China). Inert spherical particles of microcrystalline cellulose (Cellets® 90 and Cellets® 100) were purchased from Pharmatrans Sanaq AG (Switzerland). Hypromellose (Methocel E5) was donated by Colorcon Ltd. (UK). Glycerol monostearate (Imwitor 900 K) was supplied gratis by Cremer Oleo GmbH & Co. KG (Germany). Talc (Ph M) was purchased from Imerys Talc (Italy). Talc BDH was donated by BDH Chemicals (England). Silicon dioxide (Aerosil 200 Ph and Syloid AL-FP) were donated by Azelis (UK) and Grace (USA) respectively. Magnesium stearate was purchased from Acros Organics (USA). Methacrylate polymers, Eudragit® NM 30 D, Eudragit® RL 30 D and Eudragit® RS 30 D, were obtained from Evonik AG (Germany). Triethyl citrate was purchased from Sigma-Aldrich Co. (USA). Polysorbate 80 was purchased from Acros Organics (USA). Methylene blue was purchased from Acros Organics (Belgium). Hydroxyethylcellulose (Natrosol 250HX) was donated by Ashland (USA) and xanthan gum was purchased from Fluka, BioChemika (France). Isomalt (galenIQ 721) was supplied gratis by Beneo GmbH (Germany).

### Preparation of Polymer Coating Dispersions

Polymethacrylate-based copolymers, Eudragit RS/RL® 30 D and Eudragit® NM 30 D, were used in the sustained-release coatings as aqueous dispersions. The formulation compositions are described in Table I. A range of anti-tacking agents, glycerol monostearate (GMS), talc or silicon dioxide (Aerosil 200 Ph), were used in the coating formulation and the methods of preparing the anti-tacking agent dispersions were described below. To prepare the GMS dispersion, half of the required deionized water was heated to 75–80°C and GMS was added to the heated water under continuous stirring with a magnetic stirrer. Triethyl citrate (TEC) and polysorbate (Tween) 80 were added to the GMS emulsion which was stirred continuously for a further 10 min, followed by homogenisation using a rotor-stator homogenizer (Ultra-Turrax T25, IKA-Werke GmbH, Germany) at 12,000 rpm and 75–80°C for 20 min. The remaining half of the deionized water was added to the hot dispersion under continuous stirring using a magnetic stirrer and allowed to cool to 30°C. To prepare the talc or Aerosil 200 Ph dispersions, the respective anti-tacking agent was dispersed in deionized water at room temperature and homogenized at 12,000 rpm for 10 min using the rotor-stator homogenizer (Ultra-Turrax T25, IKA-Werke GmbH, Germany). TEC and Tween 80 were then added to the dispersion and homogenisation was continued under the same conditions for further 10 min.

**Table I:** Coating formulations of placebo and metoprolol succinate-containing MPs

**Coating dispersion composition**	**F1**	**F2**	**F3**	**F4**	**F5**	**F6**	**F7**	**F8**	**F9**	**F10**
Polymer used	RS/RL	RS/RL	RS/RL	NM	RS/RL	RS/RL	RS/RL	RS/RL	NM	NM
GMS, % (*w*/w) *	20	–	–	–	20	20	–	–	–	–
Aerosil 200 Ph, % (w/w) *	–	30	–	–	–	–	30	–	–	–
Talc % (w/w) *	–	–	100	100	–	–	–	100	100	100***
TEC, % (*w*/w) *	20	20	20	–	20	20	20	20	–	–
Tween 80, % (w/w) *	8	8	8	–	8	8	8	8	–	–
Water, % (w/w)	87	87	87	87	87	87	87	87	87	87
**Methods of dry powder glidant addition during coating**										
Type of glidant added	–	–	–	–	MgSt	Aerosil	MgSt	MgSt	MgSt	MgSt
Amount of glidant added**, % (w/w)	–	–	–	–	0.1	0.1	0.1	0.1	0.1	0.1
Frequency of addition (time interval, min)	–	–	–	–	30	30	15	15	15	15

* Total amount (%, w/w) based on dry polymer;

** Dry powder glidant amount (%, *w*/w) added each time based on the weight of initial cores;

***Talc Pharma M was use. All other formulations used talc grade BDH Pharma;RS/RL - Eudragit® RS/RL 30 D (9:1); NM - Eudragit® NM 30 D; TEC – triethyl citrate; MgSt – magnesium sterateSustained-release polymer coating of metoprolol succinate-containing MPs

The resultant anti-tacking agent dispersion (GMS, talc or Aerosil 200 Ph) was added to the Eudragit® RS/RL 30 D (9:1 mixture) or the Eudragit® NM 30 D dispersion under continuous stirring using a magnetic stirrer. All polymer dispersions were filtered through a 250 μm mesh sieve before coating.

### Sustained-Release Polymer Coating of Placebo MPs

Placebo microcrystalline cellulose (MCC) particles (Cellets® 100, particle size 100–200 μm) were used to evaluate the coating process outcomes of sustained-release polymer coatings using the formulations described in Table I. The coating trials were performed using 100 g starting cores in a fluidised bed coater with a Wurster insert (Mini-Glatt; Glatt GmbH, Germany). The process parameters are inlet air temperature 35–40°C (Eudragit RS/RL® 30 D) or 30–35°C (Eudragit® NM 30 D); product temperature 25–30°C (Eudragit RS/RL® 30 D) or 18–20°C (Eudragit® NM 30 D); air flow rate 18 m^3^/h; atomisation pressure 1.5 bar and spray rate 1.1–2.4 g/min. Continuous vibration was applied during all polymer coating processes using a pneumatic linear vibrator (NTS 180 NFL, Netter Vibration, Germany). Coating process was terminated when 40% weight gain was achieved.

**Fig. 1:**
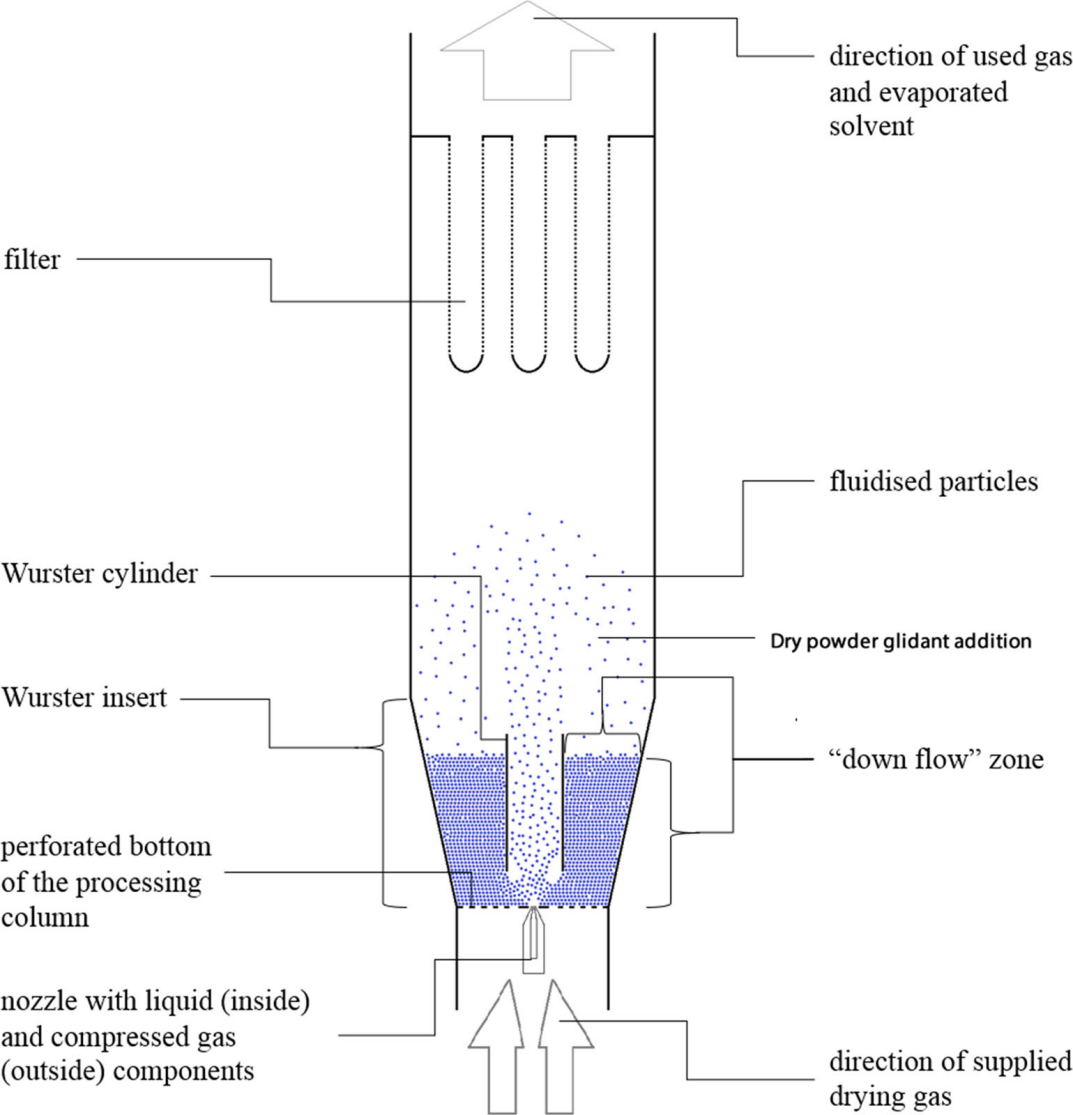
Schematic illustration of Wurster fluidised bed coating process and powdered glidant addition.

For formulations **F5** – **F10** (Table I), a novel processing method was applied, where a dry powder glidant (magnesium stearate or Aerosil 200 Ph) was periodically added (every 15 or 30 min, at 0.1 *w*/w based on starting cores for each addition) to the coating chamber through an external feeding port shown in Fig. 1. At the end of the coating process, the coated particles were dried for 20 min at 25°C *in situ*. After 10 min of drying, 1 g of silicon dioxide was added to the coating column through the external feeding port to separate particles that were stuck in the column - Non-Free Flowing Particles (NFFP, Eq. 1) and Free-Flowing Particles (FFP, Eq. 2) which were able to be discharged freely.

Sieve analysis of the discharged FFPs was conducted using a sieve shaker (AS200, Retsch GmbH, Germany) fitted with sieves of mesh sizes 90, 125, 180, 250, 355 and 710 μm. The coated particles within each size range were visualized under light microscopy (GXL3230, GT Vision Ltd., England) and the particles under the size ranges absent of agglomeration were defined as non-agglomerated particles (NAP, Eq. 3). The percentage yield of the coating trial was calculated based on the percentages of NAP and FFP **(**Eq. 4**)**.

1 $$ \% FFP=\frac{weight\ of\  FFP}{total\ weight\ of\ particles}\times 100 $$

2 $$ \% NFFP=\frac{weight\ of\ NFFP}{total\ weight\ of\kern0.5em particles}\times 100 $$

3 $$ \%\mathrm{NAP}=\frac{weight\ of\ \mathrm{NAP}}{total\ weight\ of\  FFP}\times 100 $$

4 $$ \% Yield=\frac{\%\mathrm{NAP}\times \% FFP}{100} $$

Particle size distribution analysis of the coated FFP was performed using a laser diffraction particle sizer with ASPIROS dosing at 2.0 bar and RODOS dispersing at 50 mm/s (Sympatec GmbH, Germany). This method was also used to measure particle sizes of talc and magnesium stearate.

Metoprolol succinate was layered onto Cellets® 90 cores (100 g) using the fluidised bed coater (Mini-Glatt; Glatt GmbH, Germany). The drug-loading suspension contained metoprolol succinate, hypromellose, talc (Ph M) and deionized water (22.8%, 0.6%, 4% and 72.6% *w*/w, respectively). Metoprolol succinate was dissolved in the hypromellose solution at 60°C, followed by adding and dispersing talc for 5 min using a propeller mixer (RZR 2051 control, Heidolph Instruments, Germany) at 750 rpm. The heated solution was used to increase metoprolol succinate solubility and to maximize drug concentration within the loading dispersion in order to shorten the drug loading run time. The resultant suspension was filtered through a 250 μm mesh sieve and kept at 70°C under continuous stirring with a magnetic stirrer during the drug loading process until 200% weight gain. After drug loading, three batches of polymer coating were performed using Eudragit® NM 30 D (100 g starting core, **F10**, Table I) under the process conditions described above until 300% weight gain. The polymer-coated particles were cured at 40°C for 24 h in an oven (Heratherm OMS60, Thermo Electron LED GmbH, Germany) (33).

The coating outcomes and particle size distribution of the polymer coated metoprolol succinate-containing MPs were determines the same way as the coated placebo particles. Scanning electron microscopy (SEM) was used to analyse the surface of the coated particles after the application of a 25 nm gold coating (Phenom ProX, The Netherlands). Non-destructive cone-beam X-ray computed tomography (CT; ImagiX 50 CT system, North Star Imaging Inc., USA) was performed on coated MPs using an X-ray tube with a tungsten target, 70 kV tube voltage and 140 μA tube current. A total of 1440 images were acquired (1 image every 0.25 degrees) at 2 frames per second (500 ms integration time and 3.3 μm resolution). Three-dimensional reconstruction and visualization of CT-images were performed using specialized software (myVGL, version 3.0.3, Volume Graphics GmbH, Germany; and efX-CT, version 1.9.5.12, North Star Imaging Inc., USA).

### Powder Rheology Evaluation of Coated MPs

Powder rheology tests were performed to evaluate the cohesiveness and flow properties of placebo MPs coated with Eudragit® NM 30 D (**F4,** weight gain 20%). The FFPs were immediately discharged after coating without drying and the moisture content of coated MPs was determined using a moisture analyser (MB45, Ohaus Corp., Switzerland). The densification kinetics (tapped density) of the coated MPs was determined with and without the addition of powder glidants including magnesium stearate, talc, GMS or Aerosil 200 Ph. The required amount of glidant (0.03–0.2% *w*/w based on coated MPs) was added to approximately 8.4 g of MPs. The glidant and MPs were manually mixed in a cylindrical glass bottle (40 mL, 25 mm internal diameter) for 3 min, passed through a 0.5 mm sieve before being mixed again in the same bottle for 3 min and placed into a graduated glass volumetric cylinder (10 mL, 12 mm internal diameter) fitted to a Tapped Density Tester (Copley Scientific JV1000, Copley Scientific Ltd., United Kingdom). The volume of the MPs was visualized and recorded every 3 taps until 33 taps, then at 66, 100, 1000 and 2000 taps.

The dynamic cohesive index of coated MPs was measured using a rotating-drum rheometer (GranuDrum and software GranuDrum v6.1, GranuTools sprl, Belgium). Approximately 50–60 mL of MPs were placed into a stainless-steel cylinder and rotated around its axis at an angular velocity of 2–50 rpm. The dynamic cohesive index (σ_f_, expressed as a percentage) of MPs was computed using the standard deviation from averaged steady flow (34). All measurements were made in triplicate at room temperature (20°C) or after heating to 30°C using a mini ceramic fan heater (PB-H01-UK; Pro Breeze, United Kingdom).

### Development of Metoprolol Succinate Sustained-Release Oral Suspensions Based on Coated MPs

The coated metoprolol succinate MPs (2 g) were mixed with suspending agents, hydroxyethyl cellulose (0.24% *w*/w), xanthan gum (0.24% w/w), isomalt (4.43% w/w) and silicon dioxide (Syloid, 0.04% w/w). Deionized water (20 g) was added to the mixture for reconstitution. Sedimentation stability was evaluated by measuring the height (mm) of the upper front of the suspension at predetermined time points (0, 5, 10, 20 and 30 min).

Drug release from coated MPs and reconstituted MPs suspensions (after 30 min storage) was evaluated using a USP-II apparatus (DIS 6000, Copley Scientific, UK) in 500 mL of phosphate buffer solution pH 6.8 at 37 ± 0.5°C with a paddle speed of 50 rpm. Metoprolol succinate absorbance was measured at λ 274 nm using closed-loop UV detection (T70+, PG Instruments, United Kingdom). Drug release was conducted with 12 replicates for each test.

## RESULTS

### Coating Process Outcome Evaluation Using Placebo MPs

**Fig. 2:**
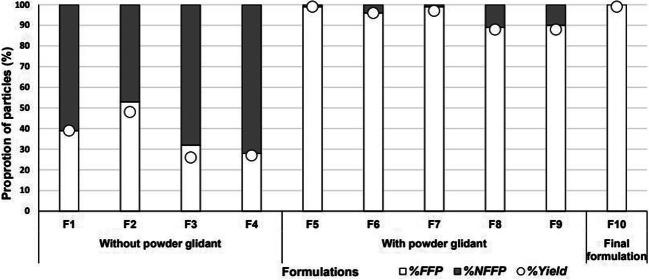
The outcomes of coating trials of formulations with and without dry powder glidant addition as listed in Table 1. % FFP: Percentage of Free Flowing Particles; %NFFP: Percentage of Non- Free Flowing Particles.

**Fig. 3:**
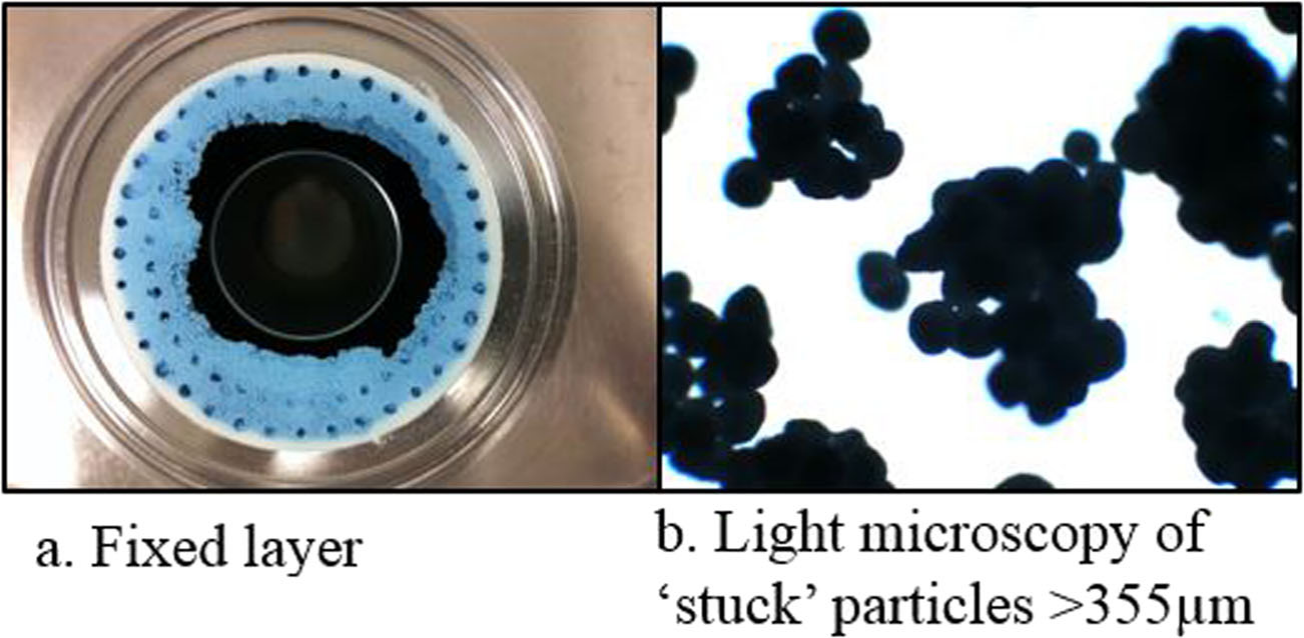
Light microscope images of particles stuck outside the Wurster cylinder (fixed layer) and agglomerated particles.

Detailed outcomes of coating process of formulations listed in Table I were evaluated using %FFP, %NFFP and %Yield **(**Fig. 2**)**. Coating placebo MCC particles (Cellets® 100) using Eudragit® RS/RL 30 D and Eudragit® NM 30 D formulations containing different anti-tacking agents - GMS, Aerosil 200 Ph and talc - in the coating liquid (**F1** – **F4**) resulted in low product yields of less than 50% (Fig. 2). For these formulations, particle agglomeration - a few particles sticking together as observed under light microscope - was low (less than 5%, data not shown) but a large portion (47–72%) of particles became stuck outside the Wurster cylinder as NFFP. Fig. 3 shows the agglomerated particles and stuck particles (NFFP) after coating. In contrast, the formulations **(F5-F9)** that received periodic addition of a small quantity of dry powder glidant, magnesium stearate (**F5 and F7-F9**) or Aerosil 200 Ph (**F6**), through the external port during coating achieved considerably high yields of over 90%, with very low rates of NFFP (less than 10%) (Fig. 2). Varying quantities of magnesium stearate (0.05, 0.1 and 0.2% every 15 min) were investigated. At 0.05%, the proportion of free flowing particles obtained was lower than 80% and at 0.2% the yield was similar to that obtained by using magnesium stearate at 0.1% (data not shown). Increasing the interval of magnesium stearate addition from 15 min to 20 and 30 min decreased the yield from approximately 97% to 85 and 78% (data not shown).

### Powder Rheology Investigation of Coated Placebo MPs

**Fig. 4:**
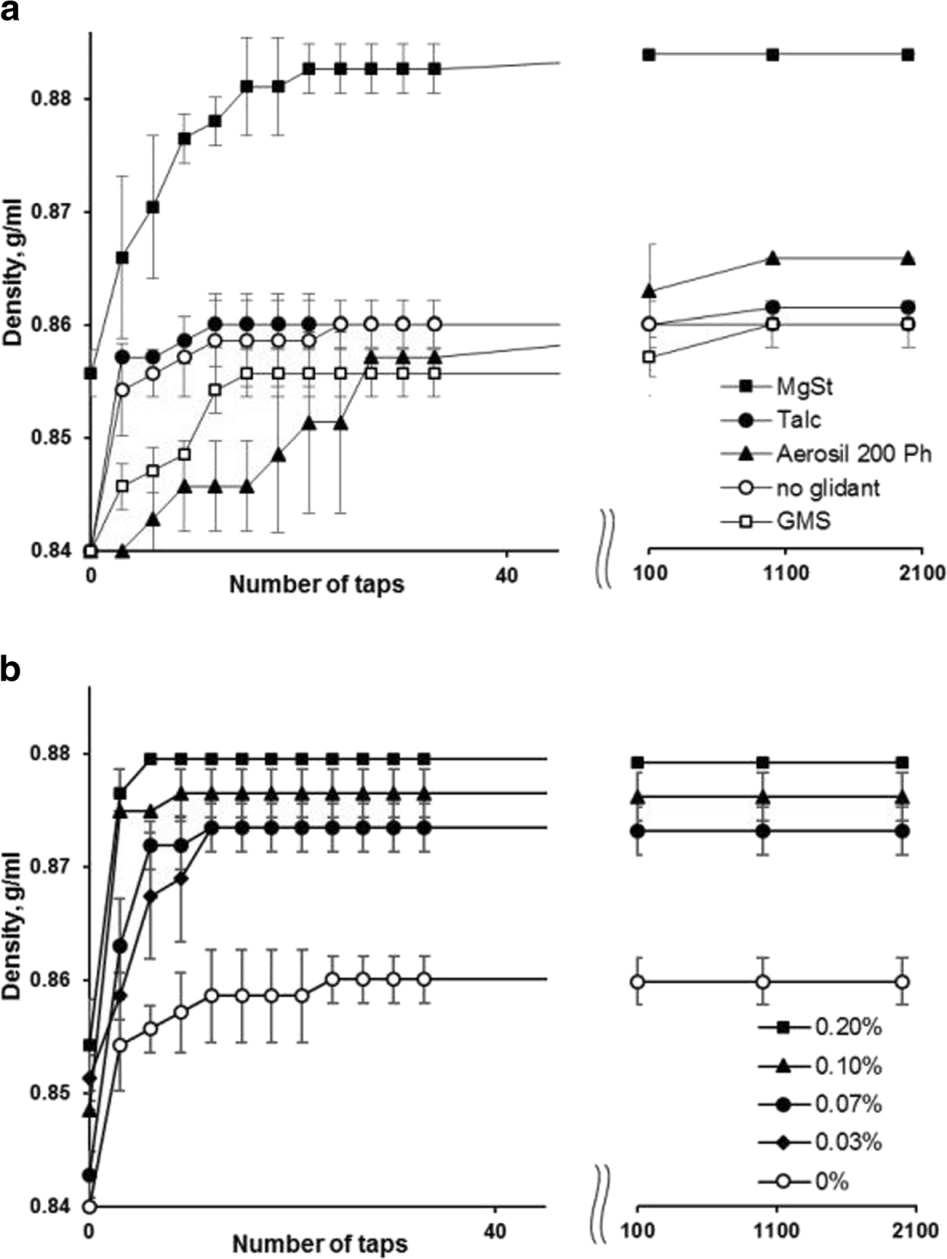
Densification kinetics of Eudragit® NM 30 D coated MPs in relation to: different glidants at concentration 0.1% (w/w) (**a**); and magnesium stearate concentrations (**B**). GMS - glycerol monostearate; MgSt – magnesium stearate.

The average particle size (D_50_) of the coated Cellets® 100 (only FFPs) after the Eudragit® NM 30 D (**F4**, WG 20%) coating was 180 μm and the moisture content was 4.7 ± 0.1% (LOD, mean ± standard deviation). Fig. 4A shows how adding different glidants at 0.1% *w*/w to the coated placebo MPs affected the powder bed densification kinetics during tapped density testing. Magnesium stearate was the most effective in increasing the tapped density of the MPs. The final tapped density of the coated MPs showed a positive relationship with the magnesium stearate concentration over the range of 0.03–0.20% *w*/w (Fig. 4B). Average particle sizes (D_50_) of talc and magnesium stearate were 12 and 10 μm, respectively. Aerosil 200 Ph had an average particle size < 1 μm (35).

**Fig. 5:**
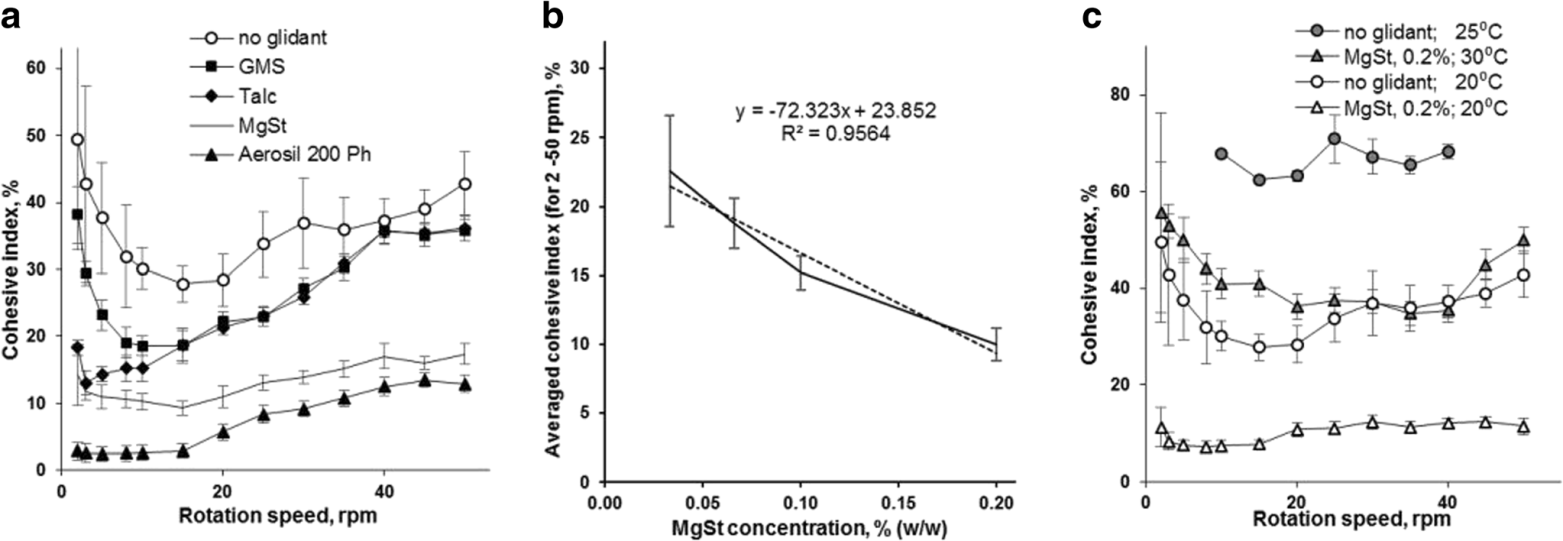
Cohesive index of Eudragit® NM 30 D coated MPs in relation to: different glidants at concentration 0.1% (w/w) (**a**); magnesium stearate concentrations (**B)** and temperature **(C)**. GMS - glycerol monostearate; MgSt – magnesium stearate.

Fig. 5A shows the dynamic cohesive index of Eudragit® NM 30 D coated MPs with and without the addition of glidants (0.1% *w*/w). Aerosil 200 Ph showed the highest effect in reducing the cohesive index of coated MPs, followed by magnesium stearate. The dynamic cohesive index of the coated MPs showed a near linear (R^2^ = 0.9564) negative relationship with the magnesium stearate concentration over the range of 0.03–0.20% (Fig. 5B). Increasing the temperature from 20°C to 30°C increased the dynamic cohesive index of the coated MPs and at both temperatures the cohesive index of MPs mixed with 0.2% w/w magnesium stearate was approximately 30% lower than without the glidant (Fig. 5C).

### Development of Metoprolol Succinate Sustained-Release Oral Suspensions Based on Coated MPs

**Fig. 6:**
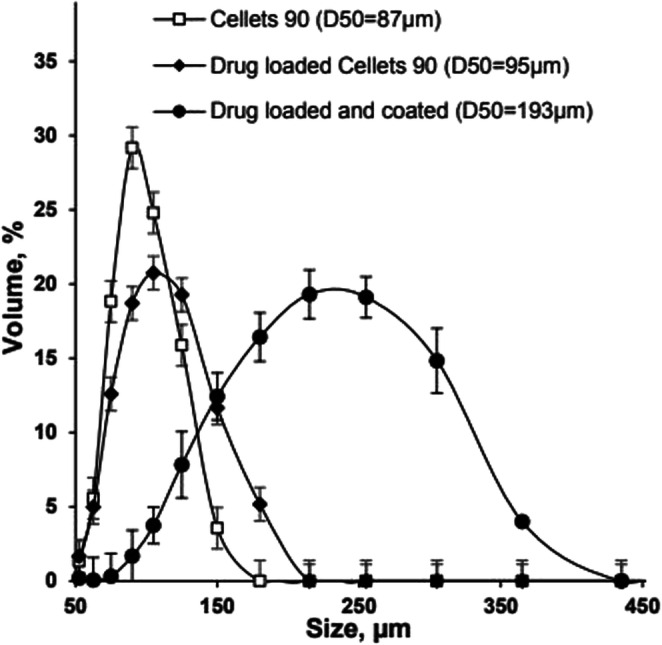
Particle size distribution measured using laser diffraction of: Cellets® 90, metoprolol succinate-loaded Cellets® 90 and Eudragit® NM 30 D coated metoprolol succinate-loaded Cellets® 90 (**F10**).

**Fig. 7:**
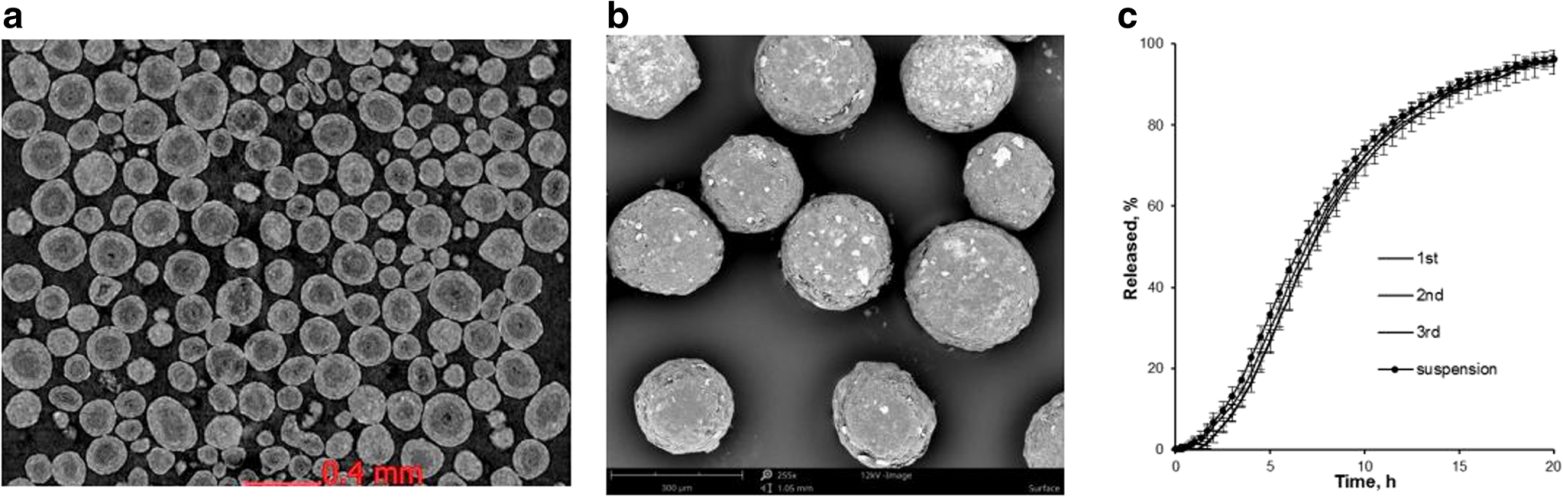
Structure of metoprolol succinate-loaded and Eudragit® NM 30 D-coated MPs: virtual cross-sections using computed tomography of MPs (**A**); SEM picture of entire MPs (**B**); and drug release profiles of three batches (1^st^, 2^nd^ and 3^rd^) of metoprolol succinate MPs coated with **F10** and after 30 min storage post reconstitution into liquid suspension (**C**).

Metoprolol succinate-loaded Cellets® 90 particles were successfully coated with Eudragit® NM 30 D aqueous dispersion **(F10)**, achieving a high product yield (99%, Fig. 2). The average particle size (D_50_) was below 200 μm (Fig. 6) and the SEM images and CT-scans of the coated MPs revealed absence of particle agglomeration (Fig. 7A and 7B). Reproducible yields (97.5–99%) and drug release profiles were achieved for the three coating batches, with drug release control up to 20 h (Fig. 7C).

Using hydroxyethyl cellulose and xanthan gum (1:1) as suspending agents at a concentration of 0.24% (*w*/w), the reconstituted metoprolol succinate MP suspension reached sedimentation stability for at least 30 min. No significant change was noted in metoprolol succinate release from MPs after reconstitution and 30 min storage in the liquid suspension compared to MPs before reconstitution (Fig. 7C).

## DISCUSSION

This study investigated an innovative concept in coating MPs (particle sizes ≤100 μm) using a Wurster fluidised bed and adding a small quantity of dry powder glidant periodically into the coating chamber throughout the coating process. Coating these small particles using aqueous dispersions of acrylic polymers - Eudragit® RS/RL 30 D and Eudragit® NM 30 D which are widely used in sustained-release coatings – resulted in low product yields due to particles becoming stuck in the processing chamber outside the Wurster cylinder. The application of the dry powder glidant successfully eliminated the stuck particles and achieved high product yield to over 95%.

To explain the positive effect of this novel approach of coating small particles, we need to understand the dynamic particle interactions and movements in the Wurster fluidised bed during coating (Fig. 1). In contrast to one-compartment fluidised beds, the Wurster cylinder divides the processing chamber into the coating/expansion and “down-flow” bed zones to improve the homogeneity of coating distribution (36). In the coating zone (within the Wurster cylinder and above the spray nozzle), particles come into contact with the atomised coating liquid droplets. The turbulent air flow moves the coated particles from the Wurster cylinder into the expanded part of the processing chamber (expansion zone), where the particles lose their velocity. Subsequently, particles move with a downward trajectory from the centre towards the perimeter of the processing chamber and settle on the top of the “down flow” bed layer outside the Wurster cylinder. From the “down flow” bed zone, particles are transferred back inside the Wurster cylinder for continued coating. Therefore, in the two-compartment processing chamber divided by the Wurster cylinder particles are in different dynamic states: in the fluidised state in the coating/expansion zone and the continuous unconstructed powder flowing state in the “down flow” zone (37).

Coating small particles can encounter agglomeration, as cohesive forces between particles are inversely proportional to the diameter of particles (37). Particle agglomeration can occur in both the coating/expansion and “down flow” bed zones during coating. Agglomeration in the coating/expansion zone is caused by the formation of liquid bridges between particles, a well reported phenomenon during the coating of small particles, especially when aqueous coating dispersions are used (31,32). Aqueous polymer dispersions based on Eudragit® RS/RL 30 D and Eudragit® NM 30 D are latex dispersions. In the coating/expansion zone, the atomised liquid droplets deposited on the particle surface loss water resulting in high polymer concentrations. The remaining water content in the polymer film acts as an additional plasticiser significantly reducing the glass transition temperature (Tg) of the polymer (38). This causes tackiness of the polymer contributing to the formation of liquid bridges. The incorporation of anti-tacking agents to the coating dispersion is the usual approach to reduce particle agglomeration in the coating/expansion zone (39), due to the reduction of the flexibility and wettability of the polymeric film decreasing tackiness (40).

In the present study, we observed that the addition of anti-tacking agents in the coating dispersion effectively prevented particle agglomeration in the coating/expansion zone and agglomeration mainly occurred in the “down flow” zone causing particles to stick outside the Wurster cylinder. The incorporation of a range of anti-tacking agents in the coating dispersion failed to solve this problem; however, the issue was resolved by strategically separating the dry powder glidant from the coating dispersion and applying directly to the “down flow” zone. The flow behaviour of particles in the “down flow” bed is affected by a number of inter-particulate forces including friction, mechanical interlocking, cohesion and liquid bridges (41). The inlet air temperature is higher than the Tg of Eudragit® NM (9°C) and plasticized (20% TEC) Eudragit® RS/RL (27°C), as such rubber-rubber interactions between particles can take place in this region (42). Liquid bridge forces may also exist due to the remaining moisture content (up to 5% *w*/w) at the particle surface. The inter-particulate forces increase with decreasing particle size and weight (43), resulting in poor particle flow and the appearance of “dead zones” in the “down flow” bed (44). This, in turn, causes a further reduction in air distribution and particle sticking in this region.

The addition of dry glidants directly in the “down flow” bed maximizes its effect in modifying surface properties of the particles including decreasing surface energy (45) and the effect of mechanofusion process (46), where the glidant particles form a mechanical barrier preventing the rubber-rubber interactions between coated particles. These surface modifications contribute to improved particle flow in the “down-flow” zone and thus preventing particle sticking in this region. The introduction of glidants into solid powder formulations to improve flowability is a well-known approach in pharmaceutical processing (47) and this study reports for the first time the innovative application of dry powder glidants during Wurster fluidised bed coating.

The reduction of surface cohesion and improvement of flow of polymer-coated particles by dry powder glidant were further investigated using powder rheology. Powdered glidants increased the tap density and decreased the dynamic cohesive index of coated MPs and magnesium stearate was one of the most effective additives, reflecting its ability to reduce the internal friction of the powder bed (34). Aerosil 200 Ph was shown to be more effective than magnesium stearate in reducing the dynamic cohesive index of coated particles but was less effective as a dry powder glidant applied during coating. It is likely that during coating, the lighter density of Aerosil 200 Ph (approx. 10 times lighter than magnesium stearate) allowed it to be blown out from the “down flow” bed to the filter housing of the processing chamber. Increasing the temperature significantly increased the dynamic cohesive index of Eudragit® NM 30 D coated particles; this can be explained by the rubbery status of the polymer. The addition of dry powder magnesium stearate significantly decreased the cohesiveness of Eudragit® NM 30 D coated particles even at an elevated temperature, contributing to its effectiveness in improving the coating process.

The technology was successfully applied to produce sustained-release MPs containing a highly water-soluble drug, metoprolol succinate, achieving reproducible product yield, particle size distribution and drug release profiles. The addition of powdered magnesium stearate during coating process could have an effect on the dissolution rate of metoprolol succinate from the coated particles. It was not possible to provide comparison of drug release from coated particles with and without the powdered glidant addition, due to the severe aggregation in the absence of powdered glidant. Larger pellets can be used to investigate and demonstrate the effects of magnesium stearate addition on drug release. The relatively small final particle size and consequently small particle weight allowed the use of low concentrations of suspending agents to produce liquid formulations (powder for reconstitution) with “in-use” stability for at least 30 min after reconstitution, allowing sufficient time for patient consumption.

The innovative particle engineering approach reported in this investigation expands the capacity of the routinely used fluidised bed in coating small particles. The application of the dry powder glidant during coating provides *in situ* stabilization of the coating process by improving particle flow in the “down flow” zone. The technology greatly improved coating production yield and reproducibility, essential for producing high quality medicinal products. Thus, it offers a reliable and scalable industrial solution for the development of sustained-release liquid medicines which could be beneficial for paediatric and older patients who cannot swallow large tablets.

## CONCLUSIONS

This is the first study to investigate a revolutionary platform for sustained-release microencapsulation using the industrial adaptable fluidised bed coating. The innovative concept of applying a small quantity of dry powder glidant periodically during coating overcame the significant challenge of particle cohesion in the “down flow” zone and achieved high product yields up to 99%. Reproducible microencapsulation of a highly water-soluble drug, metoprolol succinate, was achieved, obtaining coated MPs less than 200 μm in size with 20-h sustained drug release, suitable for producing liquid suspensions. The technology offers a first-in-class platform for the development of oral sustained-release liquid medicines providing patient-centric solutions to meet the needs of special population sub-groups, such as paediatric and older patients.
